# Effects of *Coriolus versicolor*-fermented sweet potato pulp water on yogurt: bioactive components, functional properties, and *in vitro* gut microbiota modulation

**DOI:** 10.1016/j.fochx.2025.103425

**Published:** 2025-12-18

**Authors:** Qin Cen, Jiamin Li, Zhengbin Yang, Zefen Zhu, Juan Zhou, Xinyao Huang, Huaimao Tie, Xuefeng Zeng, Likang Qin

**Affiliations:** a College of Life Sciences, Guizhou University, Guiyang 550000, China; b School of Liquor and Food Engineering, Guizhou Key Laboratory of New Quality Processing and Storage of Ecological Specialty Food, Guizhou University, Guiyang 550000, China; c Guizhou Academy of Testing and Analysis, Guiyang 550000, China

**Keywords:** *Coriolus versicolor*, Sweet potato pulp water, Yogurt；bioactive components, Gut microbiota

## Abstract

In the sweet potato starch processing industry, large volumes of organic wastewater are generated, yet their utilization remains limited. This study introduces a novel strategy for yogurt enhancement by incorporating *Coriolus versicolor*-fermented sweet potato pulp water (CV-SPPW). The effects of different CV-SPPW concentrations on yogurt's bioactive compounds, functional properties, *in vitro* gut microbiota modulation were evaluated. The results revealed that increasing CV-SPPW concentrations enriched bioactive components, including polysaccharides and triterpenoids. Notably, at a concentration of 15 %, organic acids such as lactic acid (0.6991 mg/mL) and succinic acid (0.0167 mg/mL) were maximized. Yogurt supplemented with 15 % CV-SPPW exhibited enhanced antioxidant and hypoglycemic activities. Furthermore, CV-SPPW supplementation modulated the gut microbiota by increasing the relative abundance of beneficial phyla, such as Firmicutes and Bacteroidetes, while reducing potentially harmful taxa, thereby improving microbial diversity and community structure. These findings suggest that CV-SPPW valorization provides a sustainable approach to developing functional yogurt with enhanced health benefits.

## Highlights

• A novel valorization approach of sweet potato starch wastewater *via Coriolus versicolor* fermentation.
• CV-SPPW enhanced yogurt bioactivity by enriching polysaccharides and triterpenoids.
• 15 % CV-SPPW improved yogurt antioxidant and hypoglycemic activities.
• CV-SPPW modulated gut microbiota and improved microbial diversity and balance.

## Graphical abstract

**Figure:**
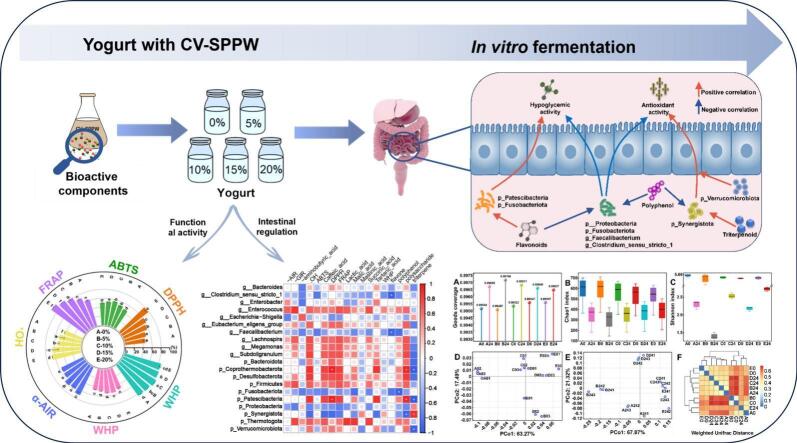

## 1 Introduction

Yogurt is a widely consumed fermented dairy product, valued not only for its unique flavor and smooth texture but also for its richness in probiotics, which contribute significantly to human health by modulating gut microbiota (Aslam et al., 2020) and enhancing immune function (Hasegawa & Bolling, 2023). However, in traditional yogurt production, chemical thickeners and stabilizers such as carrageenan and aspartame are often added to improve texture and stability. Although these additives are effective in enhancing product appearance and sensory qualities, they may have negative effects on human health, such as allergic reactions, obesity and intestinal flora imbalance(Sambu, Hemaram, Murugan, & Alsofi, 2022). Current research trends focus on the incorporation of natural bioactive compounds, for instance plant extracts and fungal fermentation products, to enhance the nutritional profile and functional properties of yogurt (Du et al., 2023).

The human gut flora is composed of trillions of microorganisms, which are widely involved in various physiological functions including nutrient absorption, energy metabolism, and immune regulation. Studies have demonstrated that dysbiosis of the gut microbiota is closely associated with the development of a range of metabolic disorders, such as obesity, diabetes, and inflammatory bowel diseases (Lan et al., 2024; Luo, Xiao, Wang, Cai, & Wang, 2024). The composition and diversity of the gut microbial community are influenced by numerous factors, including dietary habits, lifestyle, environmental exposures, and the intake of specific food components. For example, the consumption of food additives and highly processed diets has been shown to disrupt the balance of the gut microbiota (Rinninella et al., 2019). Consequently, dietary modulation of the gut microbiota through functional foods has emerged as a prominent research focus in the fields of nutrition and health, aiming to promote a beneficial microbial profile and improve host well-being.

Sweet potato pulp water (SPPW) is a major by-product generated during the industrial processing of sweet potato starch, with approximately 5 to 10 tons of wastewater produced for every ton of starch (Zhu et al., 2017). This effluent is rich in organic acids, soluble proteins, oligosaccharides, carbohydrates, indicating its high potential for resource utilization (Hu et al., 2018). However, the current utilization rate of this by-product remains low. The large-scale discharge of SPPW not only leads to significant economic losses for enterprises but also poses serious environmental pollution risks . With the growing emphasis on sustainable and circular economy development, the potential application of SPPW as a fermentation substrate for microbial cultivation has attracted increasing attention, offering a promising avenue for its considerable practical value.

*Coriolus versicolor* (CV), commonly known as cloud mushroom, is a widely studied macrofungus with well-documented medicinal properties. Its secondary metabolites are rich in a variety of bioactive compounds, including polysaccharides, triterpenoids, and flavonoids, which exhibit significant pharmacological activities such as immunomodulatory, antioxidant, anti-inflammatory, and anticancer effects (Jing et al., 2022). In recent years, increasing attention has been paid to the fermentation of CV for the production of functional metabolites, particularly due to its promising applications in the food industry (Li, Xu, & Guo, 2023). Therefore, fermenting SPPW with CV not only offers an efficient approach for valorizing this agricultural byproduct, but also enables the generation of bioactive fermentation products, providing a novel strategy and resource for the development of functional foods.

Previous studies have demonstrated that the addition of certain edible mushroom extracts can improve the physicochemical properties and sensory quality of yogurt (Bao et al., 2025). However, research on the modulatory effects of mushroom-derived fermentation products on the gut microbiota remains limited. Based on this, the present study proposes the incorporation of *Coriolus versicolor*-fermented sweet potato wastewater (CV-SPPW) into yogurt with the aim of developing a novel fermented dairy product that combines enhanced nutritional value with gut-regulatory potential.

Accordingly, the objective of this study was to investigate the effects of CV-SPPW on the bioactive compound content, antioxidant activity, hypoglycemic potential, storage stability, and the structure and function of *in vitro* gut microbiota in yogurt. This research aims to provide theoretical support for the development of nutritionally enriched, functionally enhanced yogurt products. Furthermore, the findings are expected to contribute to the broader application and value-added utilization of mushroom-derived fermentation byproducts in the food industry.

## 2 Materials and methods

### 2.1 Materials

Sweet potatoes, corn flour, and granulated sugar were purchased from Walmart Supermarket (Guiyang, China). *Coriolus versicolor* was provided by Minyuan Edible Fungi Co., Ltd. (Chongqing, China). Lactic acid bacteria yogurt fermentation powder (maltodextrin, *Lactobacillus delbrueckii* subsp. *bulgaricus*, *Streptococcus thermophilus*, *Lactobacillus acidophilus*, *Lactobacillus casei*, *Lactobacillus plantarum*) were obtained from Chuanxiu International Trading Co., Ltd. (Beijing, China). Skimmed milk powder was purchased from Yili Group Co., Ltd. (Inner Mongolia, China). All other reagents used in this study were of analytical grade unless otherwise specified.

### 2.2 Preparation of CV-SPPW and yogurt samples

The sample preparation is shown in Fig.S1 Fresh sweet potatoes were washed, chopped, and mixed with water at a material-to-liquid ratio of 1 g: 2.5 mL. The mixture was homogenized using a pulping machine (JYZ-E30, Joyoung Co., Ltd., Shandong, China) and then filtered through a 100-mesh sieve to obtain SPPW. A fermentation medium was prepared using SPPW supplemented with glucose (25 g/L), peptone (3 g/L), KH₂PO₄ (2 g/L), MgSO₄ (1 g/L), and corn flour (1 g/L). The medium was sterilized at 121 °C for 20 min and cooled to room temperature. The CV strain was activated by culturing on potato dextrose agar plates at 27 °C for 7 days, and then a fresh colony was selected for fermentation inoculation. Under sterile conditions, a 1 cm^3^ piece of activated CV culture was inoculated into the medium using a sterile inoculating loop. The culture was incubated in a thermostatic shaker as follows: static cultivation at 27 °C for 24 h, followed by shaking incubation at 170 rpm for 5 days (TCYQ, Suzhou Peiying Experimental Equipment Co., Ltd., Jiangsu, China). The resulting fermentation broth (CV-SPPW) was homogenized at 3000 rpm for 1 min (XHF-DY, Ningbo Scientz Biotechnology Co., Ltd., Zhejiang, China) and stored at 4 °C for further use.

A well-growing bacteria-free CV-SPPW was selected and homogenized at 5000 rpm/min for 5 min. CV-SPPW was added to the yogurt mixture at different volume ratios (0 %, 5 %, 10 %, 15 %, and 20 %, *v*/v). Each formulation also contained 14 % (*w*/*v*) skim milk powder and 7 % (w/v) sucrose. The mixtures were adjusted to 100 mL with drinking water, stirred until fully dissolved, and then sterilized at 90 °C for 10 min. After cooling to 42–45 °C, 0.4 % (w/v) of yogurt starter culture was added, and the mixtures were incubated at 42 °C for fermentation. Fermentation was terminated when the yogurt reached a thick consistency, with a pH of 3.9–4.2 and an acidity of 6–65°T. The yogurt was then stored at 4 °C for 24 h before further analysis.

### 2.3 Determination of bioactive components

#### 2.3.1 Determination of polysaccharide content

The polysaccharide content was determined according to the food industry standard NY/T 1676–2023. 0.5 g of sample was weighed and transferred into a microwave digestion vessel. Then, 20 mL of distilled water was added, and the mixture was homogenized thoroughly. The extraction was performed using a microwave-assisted method at 140 °C for 2 h. The extract was then centrifuged at 4000 rpm for 5 min (TGL20M, Shanghai Maijia Instrument Equipment Co., Ltd., Shanghai, China). The resulting supernatant was collected, transferred into a 100 mL volumetric flask, and diluted to volume with distilled water for further analysis. 1 mL aliquot of the extract was mixed with 1 mL of 5 % phenol solution and 5 mL of concentrated sulfuric acid. The mixture was allowed to react at room temperature for 20 min. The absorption value was measured at 490 nm wavelength (ZDM-1101, Shanghai Zhuoji Instrument Co., LTD., China). Glucose was used as the standard to construct a calibration curve for quantification.

#### 2.3.2 Determination of flavonoid content

The determination of flavonoid content was performed according to previously reported methods with slight modifications (Pramanik et al., 2025). The sample was extracted with anhydrous ethanol at a ratio of 1 mg sample to 1 mL solvent. 0.1 mL of the crude extract was mixed sequentially with 1 mL methanol, 8 mL distilled water, 0.6 mL of 5 % sodium nitrite solution, and 0.6 mL of 10 % aluminum chloride solution. After standing for 6 min, 4 mL of 1 M sodium hydroxide solution was added, and the volume was adjusted to 20 mL with distilled water. The mixture was subjected to ultrasonic extraction for 90 min (KQ-800 dB, Kunshan Ultrasonic Instrument Co., LTD., Jiangsu, China), followed by centrifugation at 10,000 rpm for 5 min. The absorbance of the supernatant was measured at 510 nm. Rutin was used as the standard, and a standard curve was established for quantification.

#### 2.3.3 Determination of polyphenol content

Polyphenol content was determined according to the Folin-Ciocalteu method (El-Nawasany et al., 2023). 1 g of sample was mixed with 30 mL of 70 % ethanol and subjected to ultrasonic extraction for 90 min. After centrifugation at 10,000 rpm for 5 min, 3 mL of the supernatant was mixed with 0.5 mL of 1 M Folin-Ciocalteu reagent and allowed to stand for 3 min. Then, 2 mL of 20 % sodium carbonate solution was added, and the mixture was shaken and incubated in the dark at room temperature for 1 h. Absorbance was recorded at 760 nm. Gallic acid was used as the standard to generate the calibration curve.

#### 2.3.4 Determination of triterpenoid content

The determination of total triterpenoids was conducted following the NY/T 3676–2020 standard. A total of 0.5 g of sample was mixed with 50 mL of aqueous ethanol and subjected to ultrasonic extraction for 1 h. The mixture was then centrifuged at 8000 rpm for 10 min. A 10 mL aliquot of the supernatant was evaporated to dryness in a boiling water bath. Subsequently, 0.1 mL of 5 % vanillin-glacial acetic acid solution and 0.8 mL of perchloric acid were added to the residue, mixed thoroughly, and incubated at 60 °C for 20 min. The reaction was terminated by cooling in an ice-water bath for 5 min, followed by the addition of 5 mL glacial acetic acid. After standing at room temperature for 10 min, the absorbance was measured at 550 nm. Oleanolic acid was used as the reference compound for constructing the standard curve.

### 2.4 Determination of water holding capacity (WHC)

An appropriate amount of sample was transferred into a centrifuge tube and subjected to centrifugation at 4000 rpm for 10 min. After centrifugation, the supernatant was carefully removed, and the precipitate was weighed. The WHC was calculated according to Eq. (1)(Qi et al., 2025).

(1) $$\mathrm{WHC}\left(\%\right)=\left(M₁-M\right)/\left(M₀-M\right)$$

Where: M₀ is the combined weight of the sample and the centrifuge tube before centrifugation, M₁ is the combined weight of the precipitate and the centrifuge tube after centrifugation, and M is the weight of the empty centrifuge tube.

### 2.5 Determination of organic acids

One gram of sample was diluted with 5 mL of water. Then, 50 μL of the diluted solution was mixed with 250 μL of 20 % acetonitrile–methanol extraction solvent, vortexed for 3 min, and centrifuged (12,000 r/min, 10 min, 4 °C). The supernatant was kept at −20 °C for 30 min and centrifuged again. The final supernatant was collected, transferred to sample vials, and stored at −20 °C. Metabolite profiling was performed using an ultra-performance liquid chromatography system (UPLC, ExionLC™ AD) coupled with a tandem mass spectrometer (QTRAP® 6500+). Chromatographic separation was achieved on an ACQUITY HSS T3 column (1.8 μm, 100 × 2.1 mm). The mobile phases consisted of water with 0.05 % formic acid (A) and acetonitrile with 0.05 % formic acid (B), with a gradient from 95:5 (A/B) to 5:95 and back to 95:5 between 0 and 12 min. The flow rate was 0.35 mL/min, the column temperature was 40 °C, and the injection volume was 2 μL. Mass spectrometry conditions included an electrospray ionization source with a temperature of 550 °C, an ion spray voltage of 5500 V in positive mode, and − 4500 V in negative mode, with a curtain gas pressure of 35 psi. In the QTRAP 6500+ system, each ion pair was monitored using optimized declustering potential and collision energy. Qualitative identification was based on a standard-built database, and quantitative analysis was conducted in multiple reaction monitoring mode.

### 2.6 Determination of antioxidant activity

A total of 1 g of each sample was mixed thoroughly with 9 mL of anhydrous ethanol and subjected to ultrasonic extraction at room temperature for 15 min. The resulting mixture was centrifuged at 12,000 rpm for 10 min. The supernatant was collected and filtered through a 0.45 μm membrane filter, and the filtrate was used for subsequent antioxidant assays. The hydroxyl radical (∙OH) scavenging activity according to the method described by Yang et al. (Yang et al., 2022). DPPH radical scavenging capacity, ABTS^+^ radical scavenging capacity and ferric reducing antioxidant power (FRAP) according to the method described by Lou et al. (Lou et al., 2024).

### 2.7 Determination of hypoglycemic activity

#### 2.7.1 α-amylase (α-Amy) inhibition rate

A 1 % (*w*/*v*) starch solution was prepared by dissolving starch in 0.1 M phosphate buffer (pH 6.9) and boiling for 15 min. An α-Amy solution with an activity of 0.1 U/mL was also prepared using the same buffer. For the assay, 0.5 mL of the sample solution was mixed with 0.5 mL of the α-Amy solution and incubated in a water bath at 45 °C for 10 min. Subsequently, 0.5 mL of the starch solution was added to the mixture and incubated for another 10 min under the same conditions. The enzymatic reaction was then terminated by adding 1.0 mL of DNS reagent, followed by heating in a boiling water bath for 5 min. After cooling, the reaction mixture was diluted with 10 mL of deionized water. The absorbance was measured at 540 nm using a spectrophotometer (Tong, Zhu, Guo, Peng, & Zhou, 2018). The α-Amy inhibitory rate was calculated according to Eq. (2).

#### 2.7.2 Α-glycosidase (α-Gly) inhibition rate

A solution of α-Gly (0.35 U/mL) and a substrate solution of 4-nitrophenyl-α-D-glucopyranoside (1.5 mM) were prepared using 0.1 M phosphate buffer (pH 6.9). In a 1.5 mL microcentrifuge tube, 50 μL of the sample solution was mixed with 100 μL of the α-Gly solution, and the mixture was incubated in a 37 °C water bath for 10 min. Subsequently, 100 μL of the 4-nitrophenyl-α-D-glucopyranoside solution was added to initiate the reaction, and the mixture was further incubated at 37 °C for 20 min. The reaction was terminated by adding 1 mL of 1 M Na₂CO₃ solution. The absorbance of the resulting solution was measured at 400 nm using a microplate reader (Tong et al., 2018). The α-Gly inhibitory rate was calculated according to the following Eq. (2).

(2) $$\text{Inhibition rate}\;\left(\%\right)=100-100\times \left(\mathrm{As}-A₁\right)/A₂$$

Where: As is the absorbance of the sample group, A₁ is the absorbance of the background control (buffer instead of enzyme), and A₂ is the absorbance of the blank control (buffer instead of sample).

### 2.8 Determination of storage stability

Storing yogurt at different temperatures (4 °C, 15 °C, and 25 °C) is intended to simulate various real-world storage conditions in order to evaluate changes in product stability. Specifically, 4 °C represents standard commercial refrigeration conditions; 15 °C simulates temperature fluctuations that may occur during transportation, retail display, or improper refrigeration; and 25 °C reflects accidental exposure to room temperature.

#### 2.8.1 Determination of pH and titratable acidity

The pH value of yogurt samples was measured using a digital pH meter (PHSJ-3F, INESA Scientific Instrument Co., Ltd., Shanghai, China). The titratable acidity was determined following Method I described in the National Food Safety Standard of China, GB 5009.239–2016: Determination of Acidity in Food.

#### 2.8.2 Determination of whey separation rate

An appropriate amount of yogurt sample was homogenized using a high-speed homogenizer at 1000 rpm for 10 min, followed by centrifugation at 10,000 rpm for 10 min. The masses of the supernatant and the precipitate were recorded separately. The whey separation rate was calculated using Eq. (3).

(3) $$\text{Whey separation rate}\left(\%\right)=M₁/M₂\times 100$$

Where: M_1_ is the mass of supernatant (g), M₂ is the mass of precipitate (g).

### 2.9 Determination of *in vitro* fermentation

#### 2.9.1 Fecal sample collection and pre-treatment

Fresh fecal samples were collected from four healthy human individuals (two males and two females) with regular dietary habits, no history of gastrointestinal disorders, and no antibiotic use within the six months prior to sample collection. A 10 g aliquot of feces was taken from each participant, pooled, and diluted tenfold (*w*/*v*) in sterile phosphate-buffered saline (PBS, 0.1 M, pH 7.0–7.4). The mixture was homogenized using a vortex mixer for 2 min and filtered through four layers of sterile gauze to remove large particulate matter. The resulting fecal slurry was stored on ice for subsequent *in vitro* fermentation experiments. This study was approved by the Human Medical Ethics Committee of Guizhou University (Approval No. HMEE-GZU-2024-7017), and informed consent was obtained from all participants.

#### 2.9.2 Preparation of fermentation medium

The fermentation medium was composed of the following components: peptone (2.00 g/L), bile salts (0.50 g/L), yeast extract (2.00 g/L), Tween-80 (2.00 mL/L), NaCl (0.10 g/L), resazurin (0.01 g/L), K₂HPO₄ (0.04 g/L), KH₂PO₄ (0.04 g/L), hemin (0.02 g/L), NaHCO₃ (2.00 g/L), vitamin K₁ (0.01 g/L), MgSO₄·7H₂O (0.01 g/L), L-cysteine (0.50 g/L), and CaCl₂·H₂O (0.01 g/L). The pH of the medium was adjusted to 7.0–7.6, followed by sterilization at 121 °C for 15 min in an autoclave.

#### 2.9.3 *In vitro* fermentation and microbiota analysis

A mixture was prepared by combining 1 mL of the fecal dilution, 9 mL of fermentation medium, and 5 % (*w*/*v*) freeze-dried yogurt samples. Each yogurt formulation was processed in three independent biological replicates. The mixtures were incubated at 37 °C under anaerobic conditions, and samples were collected at 0, 6, 12, and 24 h for further analysis.

pH: The pH of the fermentation broth was measured using a benchtop pH meter (FE-28, Mettler Toledo, Shanghai, China).

Short-chain fatty acids (SCFAs): The fermentation broth was centrifuged (10,000 r/min, 5 min), and the supernatant was mixed with 0.2 mL sulfuric acid and 0.4 mL anhydrous ether, followed by filtration through a 0.22 μm membrane. SCFAs were analyzed using an Agilent 7890 A gas chromatography system equipped with an HP-FFAP capillary column and a flame ionization detector (FID). The temperature program was set as follows: increase to 140 °C at 7.5 °C/min, then to 200 °C at 15 °C/min, and hold for 3 min. The inlet and detector temperatures were 240 °C and 300 °C, respectively.

Gut Microbiota Analysis: Genomic DNA was extracted from each biological replicate using the E.Z.N.A.® Soil DNA Kit (Omega Bio-tek, Inc., Norcross, GA, USA). The V3-V4 region of the bacterial 16S rRNA gene was amplified using universal primers 338F (5′-ACTCCTACGGGAGGCAGCAG-3′) and 806R (5′-GGACTACHVGGGTWTCTAAT-3′). To distinguish among samples, an 8-bp barcode sequence was added to the 5′ end of each primer. PCR amplification was performed using an ABI 9700 thermal cycler (Applied Biosystems, Foster City, CA, USA). Sequencing and partial bioinformatics analysis were carried out using the online platform provided by Ovison Genomics (Beijing, China; http://218.2.224.234:8888/).

### 2.10 Statistical analysis

All experiments were performed in triplicate, and the results are expressed as mean ± standard deviation (SD). Microsoft Excel was used for calculating the means and standard deviations. Statistical significance of differences among groups was assessed using one-way analysis of variance (ANOVA), followed by Duncan's multiple range test for *post hoc* comparisons (*p* < 0.05), conducted with SPSS 27. Graphs were generated using Origin 2024 software.

## 3 Results and discussion

### 3.1 Enhancement of bioactive compound content

In the previous experiment, we measured the active components in SPPW before and after fermentation (Table S1). Table 1 presents the effects of varying concentrations of CV-SPPW on the levels of bioactive compounds in yogurt. The contents of polysaccharides (from 10.41 to 20.50 mg/mL) and polyphenol (from 0.011 to 0.019 mg/mL) increased significantly with the addition of CV-SPPW (*p* < 0.05). SPPW is rich in polysaccharides and phenolic compounds, which may interact with yogurt constituents to enhance the stability and retention of bioactive substances. Moreover, during fermentation, CV secretes abundant extracellular polysaccharides that are incorporated into the SPPW matrix (Tavares et al., 2005). Additionally, bound phenolics may be hydrolyzed into more readily soluble free phenolic forms, enhancing their solubility in the SPPW phase (Li et al., 2023). Consequently, the incorporation of CV-SPPW into yogurt naturally leads to an increase in polysaccharide and polyphenol. The content of flavonoid and triterpenoid increased gradually when the CV-SPPW concentration was raised from 0 % to 10 %, but showed a decline at higher concentrations of 15 % and 20 %. At lower concentrations, these compounds are well dispersed within the yogurt matrix. However, excessive addition of CV-SPPW may lead to undesirable chemical interactions between these bioactives and other yogurt components, resulting in precipitation or degradation of certain compounds.

**Table 1:** Contents of bioactive compounds in yogurt supplemented with different concentrations of CV-SPPW.

Compounds	0 % (mg/mL)	5 % (mg/mL)	10 % (mg/mL)	15 % (mg/mL)	20 % (mg/mL)
Polysaccharide	10.41 ± 0.420^e^	12.98 ± 0.110^d^	14.36 ± 0.300^c^	17.97 ± 0.620^b^	20.50 ± 0.390^a^
Flavonoid	0.019 ± 0.001^c^	0.027 ± 0.001^b^	0.030 ± 0.002^a^	0.024 ± 0.002^b^	0.025 ± 0.000^b^
Polyphenol	0.011 ± 0.001^c^	0.017 ± 0.001^b^	0.018 ± 0.000^ab^	0.018 ± 0.000^a^	0.019 ± 0.000^a^
Triterpenoid	62.75 ± 1.870^a^	64.62 ± 2.570^a^	65.69 ± 1.210^a^	59.05 ± 0.290^b^	56.31 ± 0.670^b^

Note: Different letters within the same row indicate significant differences (*p* < 0.05).

### 3.2 Modulation of organic acids

As shown in Table 2, with the increasing addition of CV-SPPW, the concentrations of malic acid and lactic acid gradually increased, reaching their highest levels at the 15 % addition level (malic acid: 0.0020 mg/mL, lactic acid: 0.6691 mg/mL) (*p* < 0.05). Malic acid, a tricarboxylic acid cycle intermediate, enhances microbial stability in fermented products and can be converted into lactic acid by LAB, thereby improving flavor balance and yogurt palatability (Gerardi et al., 2019). In addition, the CV-SPPW may promote the growth and metabolic activity of LAB, thereby enhancing lactic acid production and improving the overall sensory quality of yogurt. However, when the addition level reached 20 %, a decline in lactic acid content was observed, which may be attributed to the inhibitory effects of excessive SPPW components on LAB growth, such as osmotic stress or the accumulation of metabolic by-products (Andersen et al., 2017). Compared to the 0 % group, all supplemented groups showed an increase in γ-aminobutyric acid content, with the highest level observed at the 5 % addition (0.047 mg/mL). This enhancement may be due to the presence of various nutrients in moderate levels of CV-SPPW, including free amino acids, vitamins, and minerals, which can stimulate LAB growth and metabolism. These factors are known to enhance the activity of glutamate decarboxylase in LAB, thereby promoting γ-aminobutyric acid biosynthesis (Nikmaram et al., 2017). In contrast, higher concentrations of CV-SPPW may introduce excess acidic substances, salts, or metabolic inhibitors into the fermentation system, potentially increasing osmotic pressure and lowering the environmental pH, ultimately reducing γ-aminobutyric acid production. The concentration of succinic acid increased from 0.0127 mg/mL (0 % group) to 0.0167 mg/mL (15 % group), followed by a slight decline at 20 % group (*p* < 0.05). This trend suggests that CV-SPPW may have modulated the microbial community structure in the yogurt, favoring the proliferation of succinate-producing microorganisms. However, at higher supplementation levels, microbial balance may have been disrupted, leading to reduced succinic acid accumulation. Notably, maslinic acid and tartaric acid were not detected in the control (0 % group) yogurt group. Tartaric acid was detected in the yogurt sample supplemented with 20 % group, with a concentration of 0.0038 mg/mL. Maslinic acid was detected in the 10 % group, at a concentration approximately seven times higher than that observed in the 5 % group. Maslinic acid is a pentacyclic triterpenic acid with various biological activities such as lowering blood sugar and anti-inflammation (He et al., 2022). Additionally, CV-SPPW supplementation introduced more caffeic acid into the yogurt. Caffeic acid is a key organic acid found in edible fungi, consistent with previous findings of its presence in mushrooms such as Agaricus bisporus and Lentinula edodes (Xiaokang et al., 2020). In summary, within an appropriate supplementation range, most organic acids in yogurt increased with the addition of CV-SPPW. However, at higher levels of addition, the content of certain organic acids declined, possibly due to the inhibitory effects of excessive CV-SPPW on microbial growth and metabolic activity.

**Table 2:** Types and concentrations of organic acids in yogurt supplemented with different concentrations of CV-SPPW.

Organic acid	0 % (mg/mL)	5 % (mg/mL)	10 % (mg/mL)	15 % (mg/mL)	20 % (mg/mL)
Malic acid	0.0009 ± 0.0001^c^	0.0017 ± 0.0000^b^	0.0014 ± 0.0001^b^	0.0020 ± 0.0002^a^	0.0017 ± 0.0001^ab^
Lactic acid	0.4692 ± 0.0584^c^	0.5409 ± 0.0252^bc^	0.6186 ± 0.0061^b^	0.6991 ± 0.0242^a^	0.4856 ± 0.0203^c^
γ-aminobutyric acid	0.0080 ± 0.0000^c^	0.0470 ± 0.0001^a^	0.0190 ± 0.0000^bc^	0.0320 ± 0.0001^b^	0.0190 ± 0.0001^bc^
Succinic acid	0.0127 ± 0.0020^c^	0.0145 ± 0.0400^b^	0.0161 ± 0. 0001^a^	0.0167 ± 0.0002^a^	0.0142 ± 0.0003^b^
Maslinic acid	–	0.0002 ± 0.0000 ^a^	0.0001 ± 0.0001 ^a^	–	–
Tartaric acid	–	–	–	–	0.0038 ± 0.0001^a^
Caffeic acid	0.0170 ± 0.0020^c^	0.0260 ± 0.0000^b^	0.0250 ± 0.0002^b^	0.0330 ± 0.0010^a^	0.0340 ± 0.0010^a^

Note: Different lowercase letters in the same row indicate significant differences between groups (*p* < 0.05). “—” indicates that the value was not detected.

### 3.3 Improvement inwater-holding capacity (WHC)

**Fig. 1:**
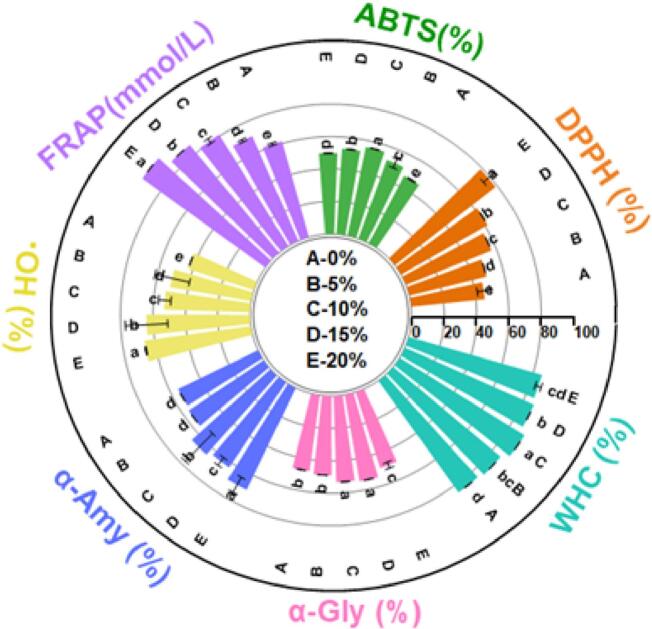
Water-Holding Capacity, Antioxidant Activity, and Hypoglycemic Potential of Yogurt. Different lowercase letters indicate statistically significant differences among the data for the same experimental parameter (*p* < 0.05). Group A represents the yogurt sample without CV-SPPW addition (0 %, *v*/v), while Groups B, C, D, and E correspond to yogurt samples supplemented with 5 %, 10 %, 15 %, and 20 % (*v*/v) CV-SPPW, respectively.

WHC is one of the key indicators for evaluating the stability and quality of yogurt, as it reflects the ability of the three-dimensional gel network to retain water (Li et al., 2023). The effect of different levels of CV-SPPW addition on the WHC of yogurt is illustrated in Fig.1. As the CV-SPPW concentration increased to 10 %, the yogurt WHC gradually improved and reached a maximum, likely due to bioactive components (*e.g.*, polysaccharides and polyphenols) interacting with yogurt proteins and lipids to form a denser gel matrix. The resulting increase in yogurt consistency ultimately enhances its WHC (Zheng, Beamer, Matak, & Jaczynski, 2018). However, when the addition of CV-SPPW exceeded 10 %, a slight decline in WHC was observed. This reduction may be due to the excessive presence of certain components in SPPW, which could disrupt the gel network or lead to phase separation, thereby weakening the structure and reducing WHC. Notably, this trend in WHC variation is consistent with the changes observed in the bioactive component content listed in Table 1.

### 3.4 Increase in antioxidant activity

Fig.1 illustrates the antioxidant activity of yogurt samples as assessed by DPPH, ABTS^+^, FRAP, and hydroxyl radical (·OH) scavenging assays. Compared to the 0 % group, the yogurt samples supplemented with CV-SPPW exhibited significantly enhanced antioxidant activities across all four assays (*p* < 0.05). Moreover, the scavenging capacity showed a positive correlation with the concentration of CV-SPPW added. Similar trends have been reported in previous studies (Bao et al., 2025). The fermentation process involving CV may alter the chemical environment of SPPW, for instance by reducing pH or modifying ionic concentrations, thereby enhancing the stability and bioactivity of certain antioxidant compounds. These changes contribute to an improved free radical scavenging capacity. In addition, various bioactive compounds present in CV-SPPW may interact synergistically with components of yogurt, such as whey proteins and probiotic metabolites, leading to further enhancement of antioxidant capacity. The enhanced antioxidant properties of the yogurt may be attributed to the synthesis of specific metabolites during the CV fermentation of SPPW, including polysaccharides, flavonoids, and polyphenol, which are known for their strong antioxidant potential (Lou et al., 2024). However, when the concentration of CV-SPPW reached 15 %, a slight decrease in ABTS^+^ radical scavenging activity was observed. This may be due to the production of certain metabolites during fermentation which exhibits antioxidant activity at lower concentrations but potentially compete with other active compounds for ABTS^+^ radicals at higher concentrations, thereby reducing the overall scavenging efficiency. In summary, the incorporation of CV-SPPW into yogurt within an appropriate concentration range significantly improves its antioxidant capacity. The extent of enhancement is positively related to the level of supplementation, thus offering a promising strategy to improve both the functional and probiotic properties of yogurt.

### 3.5 Enhancement of hypoglycemic activity

As shown in Fig. 1, the inhibition rate of α-Amy increased linearly with the addition level of CV-SPPW and reached a maximum of 69.86 ± 2.59 % at 20 % supplementation (*p* < 0.05), which is considerably higher than the inhibition level reported for other dairy-based matrices (61.31 %) (Huligere et al., 2023). This indicates that high concentrations of CV-SPPW markedly enhance α-Amy. As shown in Table S1, the polysaccharide content of CV-SPPW increased significantly after fermentation. Studies have shown that, on the one hand, polysaccharides can adsorb onto starch granules, thereby limiting their accessibility to α-Amy; on the other hand, the hydroxyl and carboxyl groups on polysaccharide chains can form hydrogen bonds with amino acid residues of α-Amy, resulting in the formation of polysaccharide–α-Amy complexes that effectively inhibit the enzyme's activity (Fu et al., 2020). As shown in Fig. 1, α-Gly activity inhibition rate increased with CV-SPPW supplementation within a certain concentration range, reaching a maximum of 54.04 ± 1.06 % at 15 % addition (*p* < 0.05). Table S1 shows that fermentation markedly elevated the levels of polysaccharides, polyphenols, and other bioactive compounds in CV-SPPW. According to Zhu et al.(Zhu et al., 2025), these molecules can reversibly bind to α-Gly *via* hydrophobic interactions, hydrogen bonding, and van der Waals forces, forming stable enzyme–inhibitor complexes and thereby suppressing catalytic activity.

### 3.6 Enhancement of storage stability

#### 3.6.1 Changes in pH of yogurt during storage at different temperatures

**Fig. 2:**
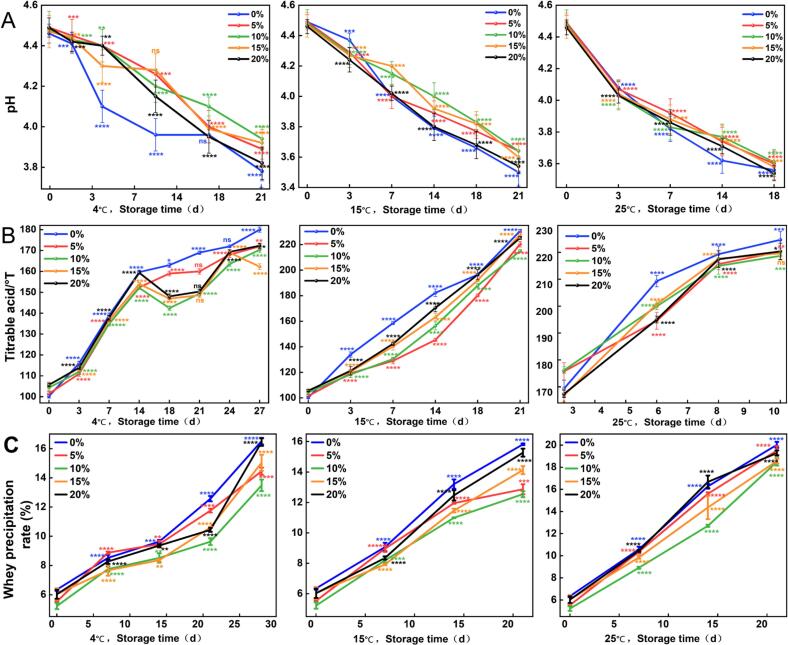
Storage stability of yogurt at 4 °C, 15 °C, and 25 °C. (A) pH. (B) Titratable Acidity. (C) Whey Separation Rate. An asterisk (_⁎_) indicates statistical significance (*p* < 0.05), a greater number of asterisks denotes a higher level of significance.

Fig. 2A illustrates the variation in pH values of yogurt samples stored at different temperatures. Under refrigeration at 4 °C, the pH of the 0 % group on day 3 was 4.41, which was comparable to the pH values of the other treatment groups. However, after day 3, the pH of the 0 % group declined more rapidly and became significantly lower than that of the other four groups. This is consistent with the trend observed in Table 2, where increasing concentrations of CV-SPPW inhibited the accumulation of organic acids. This is consistent with the pH change of the yogurt that contains added plant polysaccharides (Du et al., 2023). At 15 °C, the rate of pH decline was more pronounced than at 4 °C. With prolonged storage, all groups exhibited a significant decrease in pH. Similarly, after day 3, the 0 % group showed the fastest acidification rate among all groups. At 25 °C, changes in pH were more evident across all samples, with the 0 % group showing a markedly faster decline by day 6 compared to the CV-SPPW-supplemented groups. Comparative analysis across the three storage temperatures revealed that higher storage temperatures were associated with more rapid decreases in pH.

#### 3.6.2 Changes in titratable acidity of yogurt during storage at different temperatures

As shown in Fig. 2B, the titratable acidity of all samples increased progressively with the extension of storage time under all tested temperature conditions, which is consistent with previous findings (Bao et al., 2025). During the early stage of storage at 4 °C, there was little difference in the degree of acidity change among the groups. Collectively, after 14 days, the acidity of the 0 % and 5 % groups continued to increase, suggesting that LAB remained metabolically active, result in the accumulation of more organic acids. In contrast, the titratable acidity of the 10 %, 15 %, and 20 % groups initially decreased sharply, followed by a gradual increase. This trend indicates that high concentrations of CV-SPPW may have a temporary inhibitory effect on acid production in yogurt, potentially due to the presence of polyphenols, polysaccharides, or other bioactive compounds in CV-SPPW that interfere with LAB metabolic activity. The same results were also found in the previous report. The active substances could delay the microbial fermentation and the conversion of lactose to lactic acid, possibly inhibiting or slowing down the metabolic activity of LAB (Añibarro-Ortega et al., 2025). At 15 °C and 25 °C, the titratable acidity of all samples exhibited a nearly linear increase over time. Additionally, higher storage temperatures resulted in greater acidity at the same storage intervals. Notably, the 0 % group showed a significantly sharper increase in acidity compared to the four CV-SPPW-supplemented groups. This may be attributed to the absence of inhibitory compounds in the 0 % group, allowing microorganisms to efficiently utilize substrates such as skim milk powder and rapidly produce lactic acid, thereby accelerating acidification. In summary, elevated storage temperatures promote microbial activity, accelerating acid production. While higher concentrations of CV-SPPW exhibited an inhibitory effect on acidification during the early storage period, the differences in acidity among groups gradually diminished over time. This may be related to the regulatory effect of bioactive compounds in CV-SPPW on the growth and acid-producing metabolism of yogurt-associated microorganisms.

#### 3.6.3 Changes in whey separation rate of yogurt during storage at different temperatures

As shown in Fig. 2C, under refrigerated storage at 4 °C, the rate of whey separation in yogurts supplemented with different concentrations of CV-SPPW increased at a relatively similar pace during the initial 15 days. However, from day 15 to day 30, the rate of increase in whey separation in the 5 %, 10 %, 15 %, and 20 % groups was slower compared to the 0 %. This indicates that the yogurt matrix was more stable under low-temperature conditions, and that CV-SPPW may contain components capable of reinforcing the gel structure of yogurt during the later stages of storage, thereby reducing the rate of whey separation. These findings are consistent with earlier results on the WHC of yogurt, as shown in Fig. 1. Under storage at 15 °C, all five yogurt formulations exhibited a gradual and relatively mild increase in whey separation. In contrast, at 25 °C, the rate of whey separation accelerated considerably. Notably, on day 14 of storage at 25 °C, the 10 % group showed a significantly lower whey separation rate than the 0 % group, suggesting a stabilizing effect of CV-SPPW at elevated temperatures. As shown in Table 1, CV-SPPW contributes to the enrichment of yogurt with polysaccharides and other macromolecules, which exhibit excellent thickening and water-retention properties. These components likely enhance the integrity of the yogurt gel network, facilitating the retention of free water and thereby reducing syneresis (Mada, Duraisamy, Abera, & Guesh, 2022).

### 3.7 Regulation of gut microbiota during *in vitro* fermentation

#### 3.7.1 Changes in pH values

Changes in pH can serve as an indirect indicator of microbial fermentation activity and metabolic intensity. As shown in Table S2, all groups exhibited similar pH values at the beginning of fermentation, followed by a gradual decrease over time, which is consistent with previous reports (Zahid et al., 2023). After 24 h of fermentation, pH values decreased by 1.12, 1.83, 2.02, 2.04, and 2.28 units in the 0 %, 5 %, 10 %, 15 %, and 20 % CV-SPPW groups, respectively, indicating enhanced acid production with increasing CV-SPPW concentration. This effect is likely attributable to phenolic compounds and carbohydrates in CV-SPPW serving as carbon sources for microbial growth and metabolism, thereby accelerating organic acid formation (Tang et al., 2022). In addition, acetate has been reported to lower intestinal pH, inhibit the growth of pathogenic bacteria, and contribute to gut health (Meng et al., 2024), which is consistent with the SCFAs profiles shown in Fig. S2.

#### 3.7.2 Changes in SCFAs during *in vitro* fermentation

SCFAs are beneficial metabolites produced by gut microbiota, and their concentrations are considered important indicators of microbial activity (Meng et al., 2024). As shown in Fig. S2, acetic acid was the predominant SCFAs, followed by propionic acid and butanoic acid, consistent with previous reports identifying these acids as the major SCFAs in colonic fermentation (Zahid et al., 2023). Acetic acid increased steadily with fermentation time in all groups, with a pronounced rise between 12 and 24 h. Higher CV-SPPW supplementation resulted in greater acetate production, which plateaued at 15–20 % (124.97–125.17 mM/L). Propionic acid and butanoic acid, which are involved in lipid metabolism, insulin sensitivity, and epithelial energy supply (Wu et al., 2021), exhibited similar dynamic trends. Both showed a sharp increase after 12 h in high-dose CV-SPPW groups and reached maximum concentrations at 24 h (24.29 and 25.91 mM/L, respectively). A similar pattern was observed when phenolic- and carbohydrate-rich mango peel was added to yogurt (Zahid et al., 2023), suggesting that bioactive compounds in CV-SPPW enhance substrate availability or stimulate propionate- and butyrate-producing microbiota. At 24 h, the total concentration of seven SCFAs increased in a dose-dependent manner from 93.65 mM/L in the control group to 122.19, 166.36, 199.17, and 204.92 mM/L in the 5 %–20 % CV-SPPW groups, respectively, indicating enhanced overall microbial fermentative activity, consistent with the observed pH changes.

#### 3.7.3 Diversity analysis

**Fig. 3:**
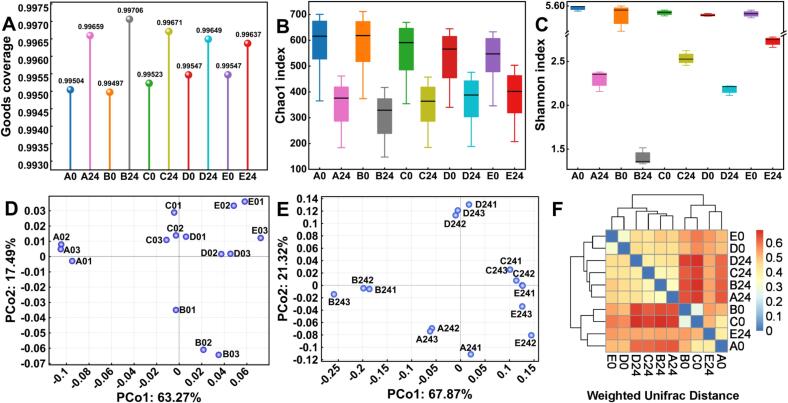
Gut microbiota diversity. (A) Goods coverage. (B) Chao1 index. (C) Shannon index. (D) PCoA-0 h. (E) PCoA-24 h. (F) Weighted UniFrac Distance. Group A represents the yogurt sample without CV-SPPW addition (0 %, *v*/v), while Groups B, C, D, and E correspond to yogurt samples supplemented with 5 %, 10 %, 15 %, and 20 % (*v*/v) CV-SPPW, respectively.

As shown in the Venn diagram (Fig.6A), a total of 219 OTUs were shared across all groups, indicating a certain degree of similarity and suggesting that the gut microbiota remained relatively stable during the experimental period. As presented in Fig. 3A, all samples exhibited Goods coverage values greater than 0.99, indicating that the sequencing depth was sufficient to accurately reflect the microbial composition of the samples. According to Fig. 3B-C, both microbial richness and diversity significantly decreased after 24 h of fermentation. Moreover, within the same fermentation period, richness and diversity declined with increasing concentrations of CV-SPPW. Interestingly, when the concentration reached 20 %, both indices began to increase again. As shown in Table 2, within a certain supplementation range, the contents of most organic acids increased with increasing CV-SPPW addition. These organic acids can reduce the pH of the gastrointestinal environment and inhibit microbial growth, thereby leading to a decrease in microbial richness and diversity. However, at higher CV-SPPW concentrations, the levels of some organic acids declined, accompanied by a rebound in microbial richness and diversity. Consistently, Renaud et al. reported that lower organic acid concentrations are associated with a more diverse gut microbiota and functions beneficial to the host (Renaud et al., 2021).

Beta diversity was assessed using principal coordinate analysis (PCoA) and Weighted UniFrac Distance to evaluate the similarity or dissimilarity of microbial communities across different samples. In PCoA plots, samples with similar microbial compositions tend to cluster together, while samples with distinct community structures are positioned farther apart (Zhang et al., 2025). As shown in Fig. 3D-E, the 0 % group was clearly separated from the four CV-SPPW-supplemented groups, indicating that CV-SPPW significantly altered the microbial community composition. In Fig. 3F, blue regions represent smaller UniFrac distances, indicating higher similarity between microbial communities, whereas red regions indicate greater dissimilarity. The most pronounced differences (in red) were observed between samples collected at 0 h and 24 h of fermentation. Hierarchical clustering analysis further supported this trend: samples collected at 24 h formed distinct clusters, while those collected at 0 h clustered together, suggesting a clear divergence in microbial composition over the fermentation period. These results imply significant shifts in microbial taxa and their relative abundance during fermentation.

#### 3.7.4 Changes in gut microbiota composition

**Fig. 4:**
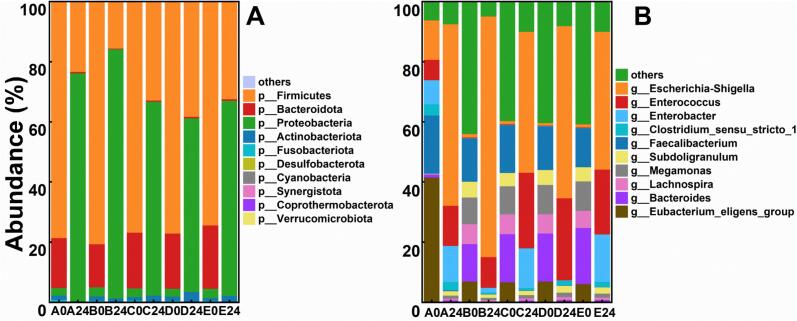
Gut microbiota composition. (A) Phylum level. (B) Genus level. Group A represents the yogurt sample without CV-SPPW addition (0 %, *v*/v), while Groups B, C, D, and E correspond to yogurt samples supplemented with 5 %, 10 %, 15 %, and 20 % (*v*/v) CV-SPPW, respectively.

As shown in Fig. 4A, the composition of gut microbiota at the phylum level was analyzed across different sample groups. At the initial stage of fermentation (0 %, 0 h), the dominant bacterial phyla included Firmicutes (78.56 %), Bacteroidetes (16.63 %), and Proteobacteria (2.51 %), which is consistent with the typical composition of the healthy human gut microbiota, indicating appropriate sample collection (Tan et al., 2022). After 24 h of fermentation, notable changes were observed. In the low-concentration group (5 %), the relative abundance of Proteobacteria increased, whereas it decreased when the CV-SPPW concentration was raised to 10–15 %. An elevated abundance of Proteobacteria in the gut is associated with microbial instability, low-grade inflammation, and even chronic colitis (Wu et al., 2021), suggesting that an appropriate increase in CV-SPPW concentration may contribute to improved gut microbial stability. It has been reported that certain members of the phylum Firmicutes promote host health by fermenting indigestible carbohydrates into SCFAs (Wu et al., 2021). When the CV-SPPW concentration reached 15 %, the relative abundance of Firmicutes was highest, likely due to the decreased intestinal pH, as acidic conditions favor their growth (Li et al., 2020). Therefore, yogurt supplemented with 15 % CV-SPPW may help optimize gut microbial composition.

At the genus level (Fig. 4B), the dominant taxa included *Escherichia-Shigella, Enterococcus, Bacteroides, Faecalibacterium, and Clostridium sensu stricto*. After 24 h of fermentation, the relative abundance of *Escherichia–Shigella* increased in all groups. Notably, in the low CV-SPPW group (5 %), *Escherichia–Shigella* remained abundant throughout fermentation, whereas its relative abundance decreased with increasing CV-SPPW concentration. This reduction may be attributed to enhanced SCFAs accumulation—particularly acetate, propionate, and butyrate, which lowers pH and thereby suppresses the growth of *Escherichia–Shigella*. This observation is consistent with the findings of Zhou et al.(Zhou et al., 2025), who reported a negative correlation between SCFAs and *Escherichia–Shigella*. Furthermore, compared with the 0 % group, CV-SPPW supplementation at 10–20 % significantly increased the relative abundance of *Enterococcus*. Xu et al. (Xu et al., 2018) demonstrated that enrichment of *Escherichia–Shigella* combined with depletion of *Enterococcus* is associated with ulcerative colitis. Therefore, the observed microbiota shifts induced by higher CV-SPPW levels may indicate a microbiota profile more closely linked to intestinal health. *Faecalibacterium*, a major butyrate producer whose metabolites serve as energy sources for colonocytes and enhance intestinal barrier function (Martín et al., 2023), reached its highest relative abundance in the 15 % group, likely due to the higher fiber content in high-CV-SPPW formulations (Godny et al., 2022). The *Clostridium sensu stricto*, which includes species capable of producing pathogenic exotoxins and is commonly regarded as a foodborne risk (Bilska, Wochna, Habiera, & Serwańska-Leja, 2024), showed a significantly lower relative abundance in the 15 % group compared with the 0 % and 20 % groups after 24 h of fermentation, indicating that a low concentration of CV-SPPW can inhibit the growth of *Clostridium sensu stricto*. Overall, yogurt supplemented with 15 % CV-SPPW exhibited the most favorable effects on modulating gut microbiota composition under *in vitro* fermentation conditions.

#### 3.7.5 Microbial functional prediction

**Fig. 5:**
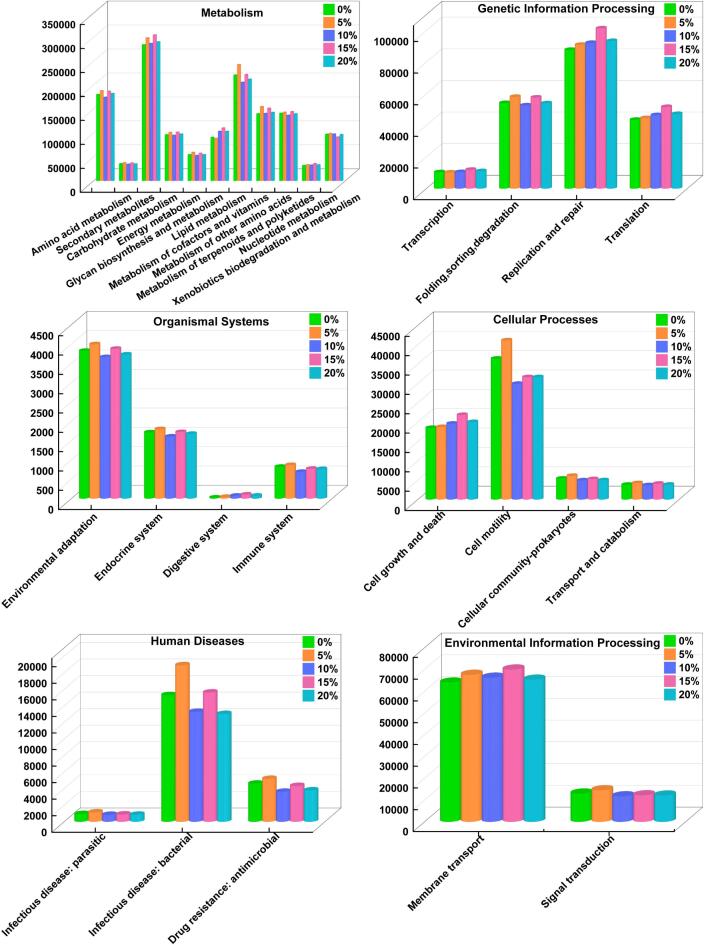
Microbial Functional Prediction.

PICRUSt2 was used to further analyze the 16S rRNA gene sequencing data and predict the functional profiles of the gut microbiota. As shown in Fig. 5, six categories were identified at KEGG level I, including Metabolism, Genetic Information Processing, Cellular Processes, Organismal Systems, Environmental Information Processing, and Human Diseases. At KEGG level II, a total of 28 functional categories were predicted. In the Metabolism category, both the 5 % and 15 % groups exhibited higher relative abundances across all KEGG level II sub-pathways compared to the 0 % group. Notably, enhancements were observed in metabolism of cofactors and vitamins and carbohydrate metabolism, suggesting that CV-SPPW supplementation may enhance energy metabolism and contribute to glycemic regulation. Within the Genetic Information Processing category, the 15 % group showed the most significant upregulation, particularly in the replication and repair pathway. In the Cellular Processes category, the 5 % group exhibited the highest enrichment, especially in cell motility, indicating that CV-SPPW may enhance microbial adaptability and the stability of the intestinal ecosystem. Previous studies have also demonstrated that the use of plant-based supplements promotes the folding changes in the cells' metabolism of energy, carbohydrates, and biotin (Petri, Mickdam, Klevenhusen, Beyer, & Zebeli, 2019). Regarding Organismal Systems, both the 5 % and 15 % groups showed notable upregulation, especially in environmental adaptation, implying improved microbial resilience to environmental stressors. Similarly, in Environmental Information Processing, both groups demonstrated increased activity, particularly in membrane transport, indicating that CV-SPPW may facilitate more efficient nutrient transport and metabolic exchange among gut microbes. However, in the Human Diseases category, functional pathways associated with infectious disease: bacterial were also elevated in the 5 % and 15 % groups. This suggests a potential risk of pathogenic bacterial enrichment, despite the overall positive impact of CV-SPPW on microbial function. In summary, CV-SPPW at 5 % and 15 % concentrations positively modulated key microbial functions, particularly those related to metabolism, adaptation, and cellular processes. Nevertheless, attention should be given to the possible enrichment of pathways linked to pathogenicity, highlighting the importance of evaluating both the functional benefits and safety aspects of CV-SPPW supplementation.

#### 3.7.6 LEfSe analysis

**Fig. 6:**
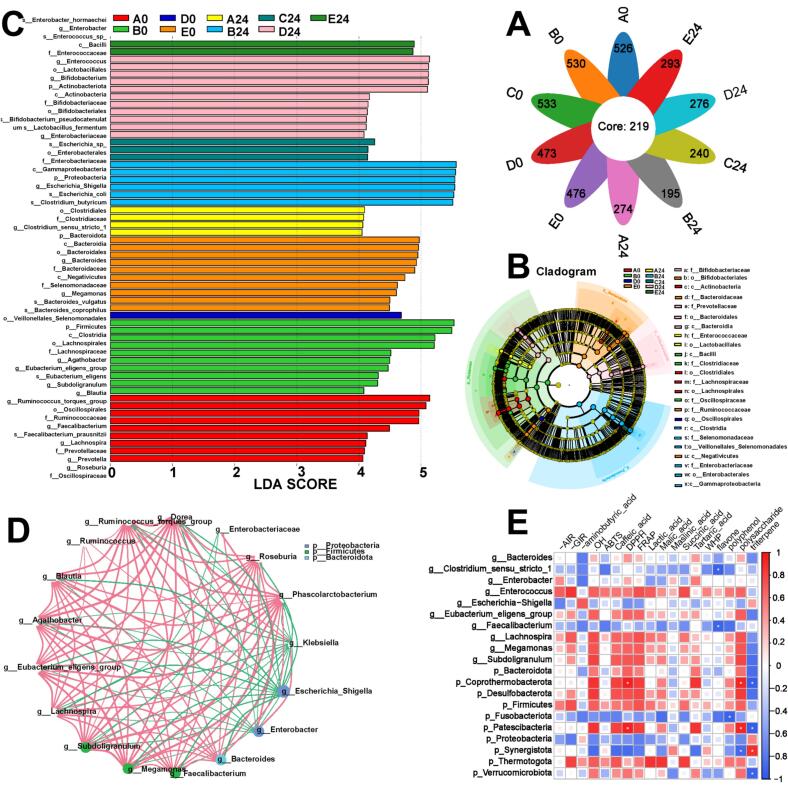
Gut microbiota composition. (A) Venn diagram. (B) Cladogram. (C) LDA Score. (D) Correlation analysis. (E) Correlation analysis of gut microbiota, red indicates a positive correlation, blue represents a negative correlation, and the color intensity reflects the strength of the correlation. Group A represents the yogurt sample without CV-SPPW addition (0 %, *v*/v), while Groups B, C, D, and E correspond to yogurt samples supplemented with 5 %, 10 %, 15 %, and 20 % (*v*/v) CV-SPPW, respectively. (For interpretation of the references to color in this figure legend, the reader is referred to the web version of this article.)

As shown in Fig. 6B, after 24 h of fermentation, the 0 % group was predominantly enriched in c_Bacilli and f_Clostridiaceae. The 5 % group was characterized by enrichment in o_Veillonellales_Selenomonadales, f_Enterobacteriaceae, o_Enterobacterales, c_Gammaproteobacteria, which are known to include potentially harmful microorganisms. In contrast, the 15 % group was significantly enriched in f_Bifidobacteriaceae, o_Bifidobacteriales, c_Actinobacteria, c_Bacteroidia, f_Enterococcaceae, o_Lactobacillales. Among these, except f_Enterococcaceae, which may contain some opportunistic pathogens, most taxa are considered beneficial microorganisms that contribute to immune modulation and gut homeostasis(Meng et al., 2024). Identifying statistically significant microbial communities through LDA Score (Fig. 6C) (Luo et al., 2024). After 24 h of fermentation, the dominant taxa in each group were as follows: 0 % group: *s_Clostridium_butyricum*, *g_Clostridium_sensu_stricto_1*, f_Clostridiaceae, o_Clostridiales. 5 % group: o_Enterobacterales, *s_Escherichia_coli*, *g_Escherichia_Shigella*, p_Proteobacteria, c_Gammaproteobacteria, f_Enterobacteriaceae. 10 % group: *s_Escherichia_sp*, *g_Enterobacteriaceae*, *s_Lactobacillus_fermentum*. 15 % group: o_Lactobacillales, *g_Enterococcus*, f_Enterococcaceae, c_Bacilli, *s_Enterococcus_sp*. 20 % group: *g_Enterobacter* and *s_Enterobacter_hormaechei*. In summary, the 10 % and 15 % groups exhibited higher relative abundances of beneficial probiotic bacteria, suggesting a more favorable microbial composition for gut health. In contrast, the 0 %, 5 %, and 20 % groups were dominated by opportunistic or potentially pathogenic microorganisms, which may pose risks to intestinal health.

#### 3.7.7 Correlation analysis of gut microbiota

A Spearman correlation analysis was performed using the top 20 OTUs ranked by absolute abundance across all samples, and the results were cross-referenced with taxonomic annotations at the phylum level. As Fig.6D, red lines indicate positive correlations, green lines indicate negative correlations, and the thickness of the lines represents the strength of the correlation. Notably, Proteobacteria exhibited significant negative correlations with Bacteroidota and several taxa within the Firmicutes phylum. Specifically, Proteobacteria are known for their high metabolic efficiency, which may allow them to rapidly consume easily accessible carbon sources, thereby limiting the growth of Bacteroidota and Firmicutes. Conversely, Bacteroidota and Firmicutes may produce antimicrobial compounds that suppress the proliferation of Proteobacteria. Interestingly, Bacteroidota showed a strong positive correlation with multiple Firmicutes taxa, indicating potential synergistic or mutualistic interactions. When carbohydrates are present as growth substrates for Bacteroidota, glycoside hydrolases and polysaccharide-degrading enzymes are released, leading to the production of short-chain fatty acids (Wu et al., 2021). Firmicutes may utilize these SCFAs as energy sources and further metabolize them to generate butyrate and other beneficial metabolites. Such positive correlations are commonly associated with a healthy and balanced gut microbiota ecosystem (Mirsepasi-Lauridsen, Vallance, Krogfelt, & Petersen, 2019). An increased abundance of Proteobacteria is often linked to gut inflammation and microbial dysbiosis, suggesting that Bacteroidota and Firmicutes may work in concert to promote SCFAs production, maintain an acidic intestinal environment, and inhibit the overgrowth of pathogenic bacteria.

#### 3.7.8 Correlation analysis between yogurt characteristics and gut microbiota

**Fig. 7:**
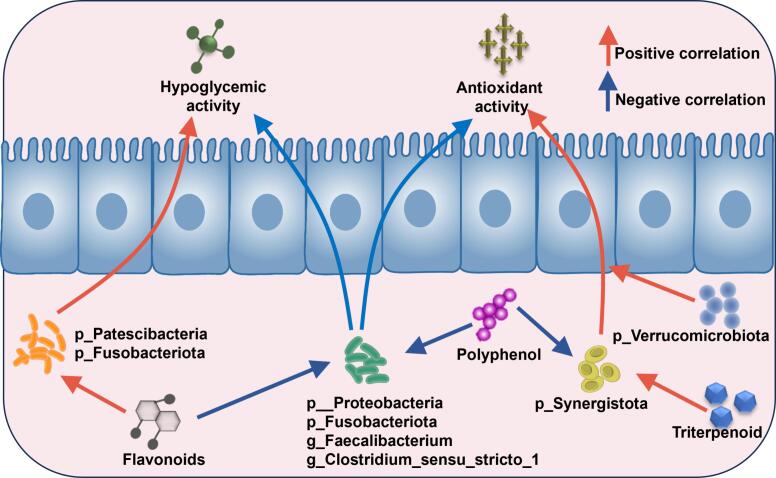
Mechanism diagram of active components regulating gut microbiota.

The correlation relationships between the active components of yogurt, organic acids, antioxidant properties, and the ability to lower blood sugar were analyzed at the species level and genus level. As shown in Fig. 6E and Fig.7, The p_Verrucomicrobiota exhibited a positive correlation with antioxidant capacity and a negative correlation with triterpenoid, suggesting that members of this phylum may contribute to antioxidant effects, potentially by consuming triterpenoid precursors during metabolism. p_Synergistota showed a positive correlation with triterpenoids and a negative correlation with polyphenols, indicating that this group may promote triterpenoid biosynthesis through symbiotic interactions, while simultaneously inhibiting polyphenol accumulation or accelerating their degradation. Moreover, p_Patescibacteria and p_Fusobacteriota were positively correlated with antioxidant capacity, polysaccharide content, and tartaric acid levels. This suggests that these taxa may enhance host antioxidant capacity either by producing antioxidant enzymes or by metabolizing polysaccharide and tartaric acid precursors. In contrast, p_Proteobacteria, *g_Faecalibacterium*, and *g_Clostridium_sensu_stricto_1* displayed negative correlations with antioxidant activity, hypoglycemic capacity, flavonoid, and polyphenol. The excessive proliferation of these microbial groups may interfere with the host's absorption or metabolism of flavonoids and polyphenols, or impair antioxidant and hypoglycemic functions through inflammatory responses.

Overall, the beneficial microorganisms *g_Lachnospira*, *g_Eubacterium_eligens_group*, p_Firmicutes, p_Bacteroidota and p_Verrucomicrobiota are positively correlated with polysaccharides and polyphenols, and also show a significant positive correlation with various indicators of antioxidant and hypoglycemic effects. The potential harmful microorganisms *g_Clostridium_sensu_stricto_1*, p__Proteobacteria, p_Fusobacteriota and p_Synergistota are often associated with inflammation and infection, and sometimes contain potential pathogenic species. They show negative correlations with active components such as flavonoids, polyphenols, polysaccharides and triterpenoids, as well as organic acids, antioxidant and hypoglycemic indicators. These results indicate that yogurt, through the enrichment of bioactive components such as polysaccharides and polyphenols through CV-SPPW, improves the antioxidant status and glucose metabolism of the body, and can also regulate the structure of the intestinal flora (enriching beneficial bacteria and inhibiting harmful bacteria).

## 4 Conclusion

This study aimed to investigate the effects of CV-SPPW on the bioactive components, functional properties, storage stability, and gut microbiota modulation of yogurt. With increasing concentrations of CV-SPPW, the contents of bioactive compounds, including polysaccharides, polyphenols, flavonoids, and organic acids in yogurt, were significantly increased. This enrichment can be attributed to the abundant nutrients and fungal-derived metabolites present in CV-SPPW, including fungal polysaccharides and bioactive secondary metabolites. Moreover, yogurt supplemented with 15 % CV-SPPW exhibited the most pronounced improvements in antioxidant and hypoglycemic activities. These enhancements are closely associated with the elevated levels of polysaccharides, polyphenols, and flavonoids, which are capable of scavenging free radicals and regulating glucose metabolism through the inhibition of carbohydrate-hydrolyzing enzymes, such as α-Amy and α-Gly. In addition, CV-SPPW improved the physical stability of yogurt during storage. Furthermore, CV-SPPW supplementation significantly increased the relative abundance of beneficial bacterial phyla, while suppressing the growth of potentially pathogenic taxa. These findings suggest a prebiotic-like effect whereby polysaccharides, phenolic compounds, and organic acids selectively stimulate the growth of health-promoting microorganisms, thereby enhancing microbial diversity and community structure. The above results demonstrate a clear logical chain for enriching bioactive components through CV-SPPW to enhance the functional activity of yogurt and regulate the intestinal flora. This provides scientific basis and a new perspective for developing functional yogurt with enhanced bioactivity and beneficial effects on intestinal health through the valorization of by-products through fungal fermentation. However, the dietary safety of CV-SPPW has not yet been systematically evaluated. Future studies should include comprehensive safety assessments and incorporate unfermented SPPW and heat-inactivated CV-SPPW as control groups, in order to more precisely distinguish matrix effects from fermentation-specific effects and to fully validate the practical applicability of CV-SPPW-enriched functional yogurt.

## CRediT authorship contribution statement

**Qin Cen:** Writing – original draft, Investigation. **Jiamin Li:** Data curation, Conceptualization. **Zhengbin Yang:** Supervision, Formal analysis. **Zefen Zhu:** Resources, Investigation. **Juan Zhou:** Visualization, Methodology. **Xinyao Huang:** Writing – review & editing, Validation. **Huaimao Tie:** Validation, Software. **Xuefeng Zeng:** Funding acquisition, Conceptualization. **Likang Qin:** Supervision, Project administration.

## Declaration of competing interest

The authors declare that they have no known competing financial interests or personal relationships that could have appeared to influence the work reported in this paper.

## Data Availability

Data will be made available on request.

## Appendix A Supplementary data

Supplementary material Image 1
