# Supplementary material for: Effects of Coriolus versicolor-fermented sweet potato pulp water on yogurt: bioactive components, functional properties, and in vitro gut microbiota modulation
Source: Food Chem X. 2025 Dec 18;33:103425. doi: 10.1016/j.fochx.2025.103425 (PMC12807823; doi:10.1016/j.fochx.2025.103425)
Supplement: Supplementary file 1 — Supplementary material [file mmc1.docx]

**Supplementary Information**


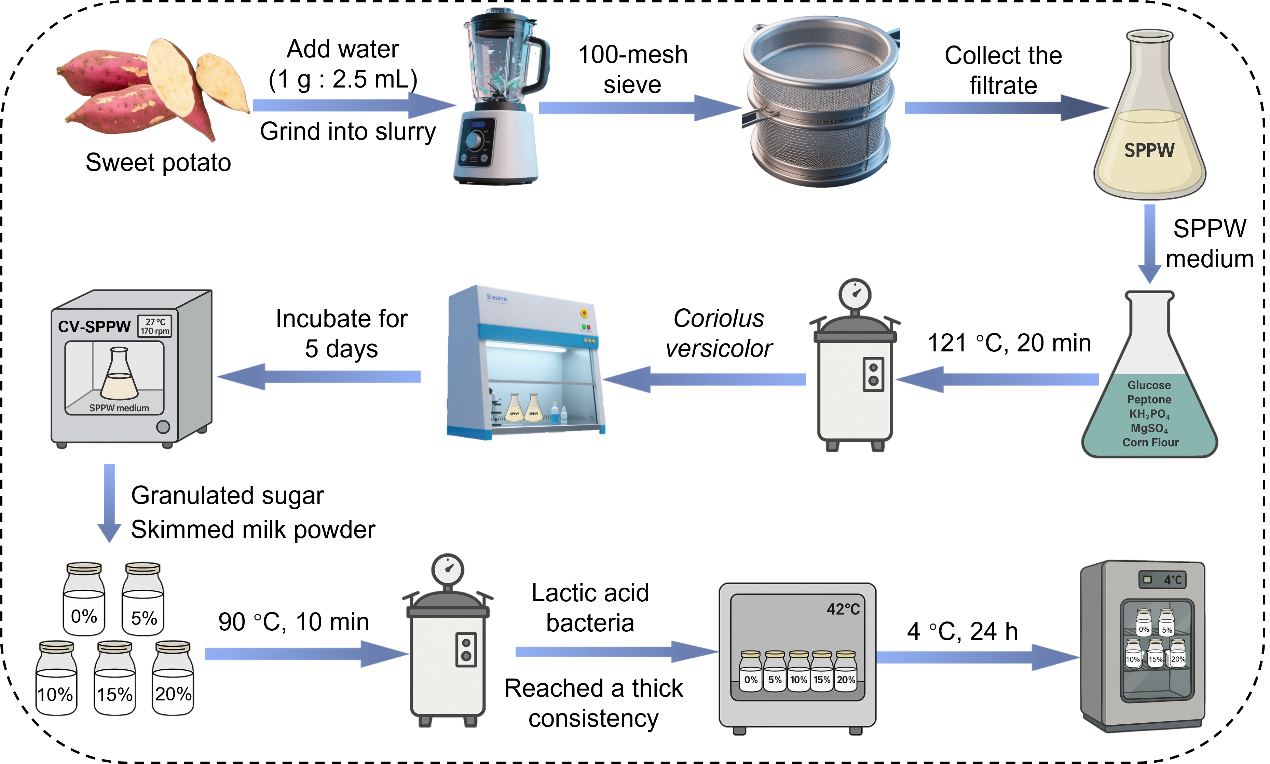
Fig.S1 Sample preparation flowchart. SPPW, sweet potato pulp water. CV-SPPW, Sweet potato pulp water fermented by Coriolus versicolor.



Fig.S2 The content of short-chain fatty acids in samples at different time points during fermentation. Different letters within each time period indicate significant differences (*p* < 0.05). Group A represents the yogurt sample without CV-SPPW addition (0%, v/v), while Groups B, C, D, and E correspond to yogurt samples supplemented with 5%, 10%, 15%, and 20% (v/v) CV-SPPW, respectively.

Table S1 The active components in SPPW before and after fermentation.

| Compounds | SPPW | CV-SPPW |
| --- | --- | --- |
| Polysaccharide (mg/mL) | 0.86 ± 0.06^b^ | 4.98 ± 0.25^a^ |
| Reducing sugars (mg/mL) | 7.02 ± 0.03^b^ | 21.76 ± 0.60^a^ |
| Flavonoid (mg/mL) | 0.03 ±0.01^b^ | 0.44 ±0.08^a^ |
| Polyphenol (mg/mL) | 0.24 ± 0.00^b^ | 2.77 ± 0.05^a^ |
| Triterpenoid (μg/mL) | 15.94 ± 0.05^b^ | 59.65 ± 0.04^a^ |
| Soluble protein(mg/mL) | 1.42 ± 0.02^b^ | 16.76 ± 0.60^a^ |

Note: SPPW, sweet potato pulp water. CV-SPPW, Sweet potato pulp water fermented by Coriolus versicolor. Different lowercase letters in the same row indicate significant differences between groups (*p* < 0.05).

Table S2 The pH values of samples at different time points during fermentation.

| Fermentation time | pH | | | | |
| --- | --- | --- | --- | --- | --- |
|  | 0% | 5% | 10% | 15% | 20% |
| 0h | 6.61±0.05^a^ | 6.84±0.28^a^ | 6.87±0.01^a^ | 6.79±0.00^a^ | 6.82±0.06^a^ |
| 6 h | 6.32±0.49^a^ | 6.1±0.04^b^ | 6.1±0.07^b^ | 5.91±0.30^b^ | 5.6±0.46^b^ |
| 12 h | 5.46±0.26^b^ | 5.17±0.22^c^ | 5.25±0.04^c^ | 5.2±0.02^c^ | 5.03±0.03^c^ |
| 24 h | 5.49±0.02^b^ | 5.01±0.00^c^ | 4.85±0.03^d^ | 4.75±0.09^d^ | 4.54±0.02^c^ |

Note: Different letters in the same column indicate significant differences (*p* < 0.05).
